# Unleashing the power of peptides in prostate cancer immunotherapy: mechanism, facts and perspectives

**DOI:** 10.3389/fphar.2025.1478331

**Published:** 2025-02-26

**Authors:** Xiaoya Li, Fang Yang, Meijing Wang, Xiaopeng Huang, Xin Zeng, Lu Zhou, Sixue Peng, Jingyi Zhang

**Affiliations:** ^1^ School of Medical and Life Sciences, Chengdu University of Traditional Chinese Medicine, Chengdu, China; ^2^ TCM Regulating Metabolic Diseases Key Laboratory of Sichuan Province, Hospital of Chengdu University of Traditional Chinese Medicine, Chengdu, China

**Keywords:** prostate cancer, peptide, immunotherapy, tumor microenvironment, immunogenic cell death

## Abstract

Prostate cancer, the second most common cancer in men, often progresses to castration-resistant prostate cancer despite androgen deprivation therapy. Immunotherapy, revolutionary in cancer treatment, has limited efficacy in prostate cancer due to its “cold tumor” nature. Peptides, with unique advantages, offer new hope. This review explores how peptide-based tumor immunotherapy can transform prostate cancer from a “cold” to a “hot” state. It modulates the immunosuppressive tumor microenvironment by regulating non-immune cells (such as cancer-associated fibroblasts, endothelial cells, and adipose stromal cells), repolarizing tumor-associated macrophages, activating NK cells, and tuning cytokines. Additionally, peptides can induce immunogenic cell death (ICD) in prostate cancer cells through ferroptosis, pyroptosis, and autophagy modulation. The review also revisits existing prostate cancer immunotherapies, including immune checkpoint blockade, CAR T cell therapy, and dendritic cell vaccines, highlighting how peptides can enhance their effectiveness and safety. Finally, two peptide-based immunotherapy strategies in the development stage, peptide-integrated Proteolysis-Targeting Chimera therapy and peptide-involved epigenomic therapy, are introduced, showing great potential for future prostate cancer treatment.

## 1 Introduction

Prostate cancer (PCa) ranks as the second most common cancer in men and is the sixth leading cause of cancer-related deaths among them (Culp et al., 2020). Androgens play a crucial role in the development and advancement of PCa. While androgen deprivation therapy (ADT), often combined with supplementary treatments like radical prostatectomy and radiotherapy, can effectively manage and slow down the disease’s progression in its early stages (Wala et al., 2023), the development of castration resistance is an inevitable outcome. As a result, a significant proportion of patients will eventually progress to castration-resistant prostate cancer (CRPC) (Nguyen et al., 2015).

Immunotherapy has revolutionized cancer treatment, providing lasting responses and wide-ranging applicability across multiple cancer types (Zhang and Zhang, 2020). By modulating autoimmune responses, enhancing antigen presentation, and disrupting inhibitory tumor microenvironment (TME), this groundbreaking approach strengthens the immune system’s ability to detect and eliminate cancer cells (Chow et al., 2022). Notably, immunotherapy has achieved significant advancements in the treatment of certain genitourinary tumors (Mehta et al., 2017).

Despite its promise, immunotherapy in PCa has yet to deliver transformative outcomes, largely because PCa is classically considered a “cold tumor” (Bhatia et al., 2023). When compared to highly immunogenic tumors such as melanoma, PCa poses unique challenges for immunotherapy (Topalian et al., 2019). Its TME often displays immunosuppression, marked by a preponderance of regulatory T (Treg) cells, myeloid-derived suppressor cells, and M2 macrophages, which facilitate immune evasion (Kwon et al., 2021). Additionally, prostate tumors generally harbor fewer neoantigens due to their relatively low mutational load, thereby reducing their immunogenicity. Consequently, PCa frequently exhibits resistance to immunotherapeutic strategies (Subudhi et al., 2020).

Since the introduction of insulin almost a century ago, more than 80 peptide drugs have reached the market for a wide range of diseases, including diabetes, cancer, osteoporosis, and multiple sclerosis (Muttenthaler et al., 2021). Peptides represent a unique class of pharmaceuticals comprising amino acids, with molecular weights spanning from small molecules to proteins (Abbas et al., 2019). The scientific community has increasingly recognized numerous advantages of peptides over small molecules and biological agents. These benefits encompass lower production costs, enhanced tumor tissue penetration, decreased immunogenicity and toxicity, straightforward target binding, versatile sequence selectivity, and remarkable efficacy (Liu et al., 2019). Many peptides utilized in cancer immunotherapy are derived from the functional domains of proteins and exhibit specific activities such as receptor binding (Achilefu et al., 2002), responsiveness to stimuli (Gupta et al., 2023), cell penetration (Song et al., 2019), and modulation of cell signaling pathways (Hanold et al., 2017). Notably, peptides have the potential to augment immune responses in the context of tumor immunotherapy (Sun et al., 2018).

In this review, we discuss how peptide-based tumor immunotherapy transforms PCa from a “cold” to a “hot” state by modulating the TME of PCa and inducing ICD in PCa cells, thereby enhancing the response of PCa to immunotherapy. Subsequently, we will review the existing modifications in PCa tumor immunotherapy that have entered clinical trials but have yet to achieve substantial breakthroughs, along with the potential improvements peptides can offer in this regard. Finally, we introduce two areas of peptide-based immunotherapy for PCa that are currently in the development stage.

## 2 Transforming the immunosuppressive prostate cancer tumor microenvironment: from “cold” to “hot” with peptides

**FIGURE 1:**
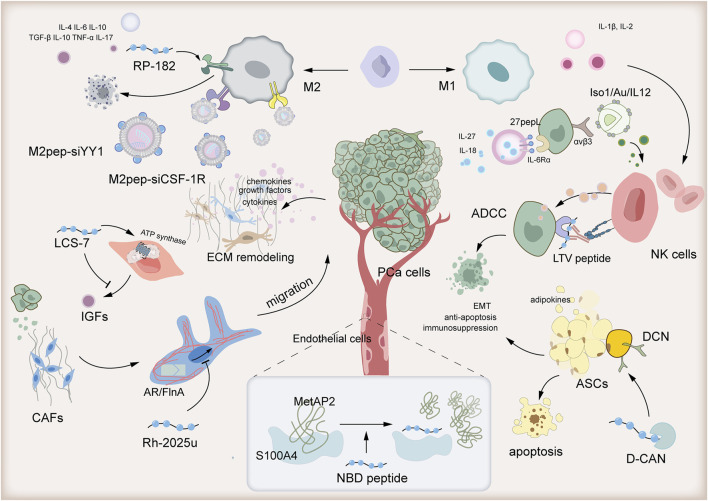
Peptide-based tumor immunotherapy transforms the immunosuppressive prostate cancer tumor microenvironment from “cold” to “hot.”

The TME of PCa is distinguished by T cell exhaustion and immunosuppressive activity of Treg cells, M2 macrophages, and quiescent NK cells (Kang et al., 2022). Furthermore, the intricate crosstalk between PCa cells and stromal cells, including Cancer-associated fibroblasts (CAFs), endothelial cells, and adipose stromal cells (ASCs), contributes to the progression of PCa (Heidegger et al., 2022). In the following, we will introduce the utilization of peptides to modulate the functions of these cells, thereby improving the immunosuppression within the TME of PCa Figure 1.

### 2.1 Orchestrating the non-immune cells in the tumor microenvironment with peptides

#### 2.1.1 Peptide mediation in cancer-associated fibroblast-induced extracellular matrix remodeling

Cancer cells recruit CAFs, which are crucial in shaping the malignant TME. Activated CAFs facilitate tumor cell growth, invasiveness, and drug resistance by depositing extracellular matrix (ECM) and secreting cytokines, chemokines, and growth factors (Gascard and Tlsty, 2016). A seemingly straightforward therapeutic approach would be to directly target and eliminate CAFs. However, recent studies have shown that depleting αSMA (+) fibroblasts paradoxically heightens the risk of tumor invasion or migration (Özdemir et al., 2014). To address this concern, peptide-based tumor immunotherapy research has shifted its focus to inhibiting the recruitment and activation of CAFs by PCa cells, with the goal of functionally modulating CAFs instead.

In response to androgen stimulation, PCa cells recruit and activate CAFs, which significantly enhance the size of PC organoids by remodeling the ECM (Di Donato et al., 2015). The stapled peptide Rh-2025u, derived from the androgen receptor (AR), disrupts the interaction between androgen-bound AR and Filamin A (FlnA), thereby preventing the formation of the AR/FlnA complex. By inhibiting this complex, Rh-2025u further suppresses the migration of CAFs (Di Donato et al., 2021a). Specifically, the AR/FlnA complex, in conjunction with integrin β1, forms a ternary complex that regulates focal adhesion kinase, paxillin, and Rac, collectively orchestrating the migration of prostate CAFs (Di Donato et al., 2021b). Rh-2025u effectively disrupts the downstream signaling of this ternary complex, thus inhibiting the migration process of prostate CAFs (Di Donato et al., 2021a).

Cancer cells’ phenotypes and molecular functions are intrinsically tied to signals originating from outside the cell, particularly through interactions with ECM (Winkler et al., 2020). These ECM components undergo modifications by binding with soluble factors, including growth factors and other ECM-associated proteins (Hynes, 2009). The surface receptors on cancer cells engage with ECM components and ECM-bound factors, facilitating cell adhesion and signaling, which in turn regulate a wide array of processes such as proliferation, differentiation, migration (Gonzalez and Medici, 2014). In the TME of PCa, prostate stromal cells, especially CAFs, secrete insulin-like growth factors (IGFs), thereby promoting extracellular matrix remodeling (Goel et al., 2006). Normally, IGF-IR expression is observed in prostate epithelial cells and PCa cells (Ryan et al., 2007). Increasing evidence suggests that IGFs play a crucial role in the development of PCa, including in the process of epithelial-mesenchymal transition (Kawada et al., 2006). LCS-7, a peptide mycotoxin derived from leucinostatin (LCS)-A, binds to the crucial carboxylate group of Glu59 on the mitochondrial ATP synthase subunit c, effectively inhibiting ATP synthase activity. This leads to mitochondrial dysfunction and subsequently impedes IGFs synthesis. Studies have demonstrated that LCS-7 significantly retards the growth of mouse xenograft tumors (Ohishi et al., 2020).

#### 2.1.2 Peptide regulation of angiogenesis through endothelial cells

Endothelial cells play a crucial role as a non-immune component within the TME. They contribute to the development and expansion of tumor blood vessels by secreting angiogenic factors like Vascular Endothelial Growth Factor (VEGF) and engaging in interactions with macrophages and tumor cells, ultimately influencing the migration and invasion of tumor cells (Arab et al., 2023).

The synthetic NBD peptide, which mimics the S100A4-binding domain of MetAP2, has been demonstrated to regulate the expression of genes related to angiogenesis and suppress capillary formation in murine endothelial cells (Takenaga et al., 2021). MetAP2, an effector protein of S100A4 known to be involved in angiogenesis, binds to S100A4 in a calcium-dependent manner. When calcium binds to S100A4, it undergoes a significant conformational change, forming a hydrophobic binding pocket that can interact with MetAP2 (Ochiya et al., 2015). The NBD peptide effectively blocks this binding pocket, thus inhibiting the interaction between S100A4 and its effector proteins, including MetAP2 (Takenaga et al., 2021).

#### 2.1.3 Peptide modulation of epithelial-mesenchymal transition via adipose stromal cells

ASCs secrete adipokines, including mitogenic factors, that foster a tumorigenic microenvironment through paracrine signaling. These adipokines facilitate processes such as tumor epithelial-mesenchymal transition (EMT), suppress the immune system, and counteract apoptosis, all of which contribute to cancer progression (Saha et al., 2023).

To effectively target and eliminate ASCs, hunter-killer peptides such as D-CAN have been devised (Su et al., 2019). D-CAN is composed of two essential parts: an ASC-binding domain (peptide CSWKYWFGEC) and a pro-apoptotic fragment (Daquinag et al., 2017). Notably, the ASC-binding peptide CSWKYWFGEC selectively binds to decorin (DCN), a unique marker expressed on the ASC surface (Daquinag et al., 2017). Consequently, this peptide serves as a potent tool for delivering ASC-targeted therapy, with the aim of inhibiting obesity-associated EMT and progression of PCa.

### 2.2 Repolarizing tumor-associated macrophages with peptides

Tumor-associated macrophages (TAMs) play a central role within the TME, and their reprogramming is intricately linked to the transformation of this environment (Xiang et al., 2021). TAMs can be broadly classified into M1 and M2 phenotypes. M1 TAMs display antitumorigenic characteristics, secreting cytokines like IL-12 to directly eliminate tumor cells and recruiting cytotoxic T cells for indirect antitumor effects (Li et al., 2021). In contrast, M2 TAMs, which are associated with tumor immunosuppression and angiogenesis, proliferate with tumor growth and dominate the immunosuppressive TME. Their immunosuppressive activities are mediated by the release of factors such as IL-6, contributing to resistance to immunotherapies (Chen et al., 2021). A study revealed that cathelicidin-related antimicrobial peptide (CRAMP) derived from PCa facilitates the differentiation of immature myeloid progenitors into macrophages and further polarizes them into the M2 phenotype within the TME (Cha et al., 2016). This aligns with the notion that PCa is characterized as a “cold” tumor. Consequently, a promising therapeutic strategy for treating tumors involves targeting M2-like TAMs by inhibiting their recruitment, altering their phenotype, and disrupting their functional activities.

RP-182 is a synthetic, 10-amino acid amphipathic analog of host defense peptides, meticulously crafted to elicit a conformational shift in the CD206 mannose receptor present on M2-like TAMs (Jaynes et al., 2020). Upon activation of this receptor in both human and murine M2-like macrophages by RP-182, it initiates a cascade of cellular events encompassing endocytosis, phagosome-lysosome fusion, and autophagy. As a result, RP-182 reprograms these M2-like TAMs into an antitumor M1-like phenotype, thereby augmenting both innate and adaptive antitumor immune responses. This reprogramming also enhances the phagocytosis of PCa cells by the modified TAMs (Jaynes et al., 2020).

Moreover, research efforts have centered on leveraging M2pep for the targeted delivery of CSF-1R siRNA (Sun et al., 2023b). The CSF-1/CSF-1R signaling axis plays a pivotal role in dictating the polarization state of TAMs. By employing small interfering RNA (siRNA) to knock down CSF-1R expression, studies have demonstrated successful reprogramming of TAMs, shifting them from the M2 phenotype to the M1 phenotype (Li et al., 2020). To enhance the precision of cellular delivery, M2pep was integrated into amphiphilic cationic β-Cyclodextrin (CD) nanocarriers for the encapsulation of CSF-1R siRNA (Sun et al., 2023b).

Lastly, research has shown that utilizing an M2-targeting peptide-modified liposome carrier for YY1-targeted therapy in M2 macrophages is highly effective in inhibiting PCa cell lung metastasis and exhibits synergistic antitumor effects when combined with PD-1 blockade (Chen et al., 2023). Yin Yang 1 (YY1), a highly conserved C2H2 zinc finger nuclear transcription factor that is overexpressed in M2 macrophages within PCa tissues, plays a crucial role in this process (Cho et al., 2018). During M2 macrophage polarization, the IL-4/STAT6 signaling pathway upregulates YY1, leading to the formation of a liquid-liquid phase separation (LLPS) structure. Within this LLPS, YY1 interacts with transcriptional cofactors such as p300, p65, and CEBPB to enhance IL-6 expression in macrophages (Tsang et al., 2020). To specifically target and silence YY1 in M2 macrophages, researchers have synthesized the M2 macrophage-targeting peptide M2pep, identified through a subtractive phage display approach, onto a liposome carrier loaded with YY1 siRNA (M2pep-siYY1) (Chen et al., 2023).

### 2.3 Activation of natural killer cells with peptide

Natural killer (NK) cells are pivotal in inhibiting cancer progression by eradicating mutant cells through death receptors and cytotoxic granules. However, within the TME, these NK cells frequently exhibit exhaustion (Jia et al., 2023). Hence, ongoing research in PCa is focused on developing peptide-based immunotherapies aimed at reversing NK cell exhaustion and enhancing their cytotoxic functions.

A novel antibody has been created that harnesses antibody-dependent cellular cytotoxicity (ADCC) to activate NK cells. This antibody features a specific cancer cell-binding peptide, LTVSPWY (LTV peptide), fused to the Fc domain of human IgG1. Identified through peptide phage display libraries, the LTV peptide exhibits high affinity and specificity for binding to PCa cells (Sioud et al., 2015). Preliminary findings suggest that this peptide-Fc fusion protein enhances NK cell activation and demonstrates ADCC against cancer cells. Furthermore, it boasts improved tissue penetration and reduced toxicity due to its targeted delivery, making it a promising antitumor agent that could potentially overcome the limitations of traditional monoclonal antibodies, which may not elicit a response in all patients (Sioud et al., 2015).

Moreover, in a study, an innovative strategy was employed to enhance the therapeutic index of interleukin-12 (IL-12) by targeting its delivery to tumors (Gasparri et al., 2019). IL-12 is a highly immunostimulatory cytokine capable of activating NK cells, but its systemic administration is hampered by toxicity concerns (Berraondo et al., 2018). To address this challenge, the researchers utilized a tumor-targeting peptide, Iso1, which recognizes the αvβ3 integrin overexpressed on tumor vessels and cells. They conjugated Iso1 to gold nanoparticles loaded with IL-12 (Iso1/Au/IL-12) and evaluated this nanoformulation in murine models of PCa. The findings revealed that this sophisticated delivery system facilitated the administration of minuscule yet therapeutically effective doses of IL-12 directly to PCa cells, triggering the activation of NK cells (Gasparri et al., 2019).

### 2.4 Modulating cytokines in the tumor microenvironment using peptides

Cytokines play pivotal roles in orchestrating immune responses and driving the progression of PCa (Mao et al., 2021). Pro-inflammatory cytokines, such as IL-1β and IL-2, augment the infiltration of cytotoxic T lymphocytes (Li and Ruffell, 2022). Conversely, in CRPC, levels of anti-inflammatory cytokines including IL-4, IL-6, and IL-10 are elevated, while TNF-α and IL-17 upregulate PD-L1 expression, further intensifying immunosuppression (Cheng et al., 2024). Additionally, TGF-β and IL-10 dampen autoimmune responses (Wise et al., 2000). Thus, modulating these cytokines emerges as a critical approach in immunotherapy for PCa.

IL27pepL, also known as 27pepL, is a novel derivative of the cytokine IL-27. This derivative is created by modifying the C-terminus of IL-27 with a specific short peptide called pepL (LSLITRL). PepL, consisting of the amino acid sequence Leu-Ser-Leu-Ile-Thr-Arg-Leu, has the unique ability to bind to the interleukin-6 receptor α (IL-6Rα) that is frequently overexpressed in tumor cells (Figueiredo Neto et al., 2020). By utilizing peptides, such as pepL, that bind to receptors upregulated in tumor cells, such as IL-6Rα, researchers aim to directly target the cytokine to tumor tissue and enhance the bioactivity of IL-27 (Figueiredo et al., 2021). IL-27 plays a central role in boosting interferon-gamma (IFNγ) production by inducing the T-box transcription factor 21 (Tbx21) and fostering T helper 1 (Th1) cell differentiation (Takeda et al., 2003). Meanwhile, IL-18 potentiates the immune system by stimulating the proliferation of various immune effector cells and enhancing major histocompatibility complex (MHC) class I expression (Tse et al., 2011). Remarkably, the sequential administration of these cytokines—first 27pepL, followed by IL-18—exerts a synergistic effect, significantly amplifying their bioactivity and efficacy against prostate tumors (Figueiredo et al., 2021). This strategy not only maximizes the therapeutic potential of each cytokine but also offers a versatile approach to incorporating gene delivery and recombinant cytokines as tools to enhance anti-cancer treatment efficacy.

A recent study has demonstrated that peptide 5a, sourced from chromogranin A and designed to target αvβ6 and αvβ8 integrins, exhibits remarkable inhibitory effects on TGFβ activation in PCa cells that overexpress these receptors (Monieri et al., 2023). Both αvβ6 and αvβ8 integrins facilitate TGFβ activation by interacting with the RGD sequence present in the latent, inactive LAP (latency-associated peptide)-TGFβ complex. Peptide 5a disrupts this interaction by specifically binding to the binding site of the LAP/TGFβ complex, thereby impeding TGFβ activation (Van Aarsen et al., 2008). More specifically, the RGDL motif and stapled alpha-helix structure of peptide 5a mimic the binding interactions with the proTGFβ1/αvβ6 complex, effectively preventing TGFβ from being activated (Nardelli et al., 2019). This dual-targeting mechanism enhances the specificity, effectiveness, and therapeutic promise of peptide 5a. By zeroing in on tumors and inhibiting immunosuppressive cytokines like TGFβ, peptide 5a has the potential to augment antitumor immunity, presenting a fresh approach to modulating cytokines within the TME (Monieri et al., 2023).

However, TGF-β signaling is intricate. In its SMAD pathway, TGF-β binds to type II receptors, leading to the phosphorylation of downstream R-SMADs (R-SMAD2 and R-SMAD3) (Cao and Kyprianou, 2015). These phosphorylated R-SMADs form complexes with cytosolic SMAD4 and translocate to the nucleus, regulating gene expression that results in G1 cell cycle arrest and apoptosis in early-stage PCa cells (Jones et al., 2009). Beyond the SMAD pathway, various non-SMAD pathways, such as MAPK, c-Src, m-TOR, RhoA, RAS, PI3K/Akt, and protein phosphatase 2A/p70s6K are also regulated by TGF-β. Consequently, TGF-β’s role in PCa cells is complex, exhibiting tumor-inhibitory effects in early stages and tumor-promoting effects in advanced stages, along with its immunosuppressive function (Collazo et al., 2014). Therefore, solely inhibiting the tumor-promoting actions of TGF-β may pose challenges in achieving a therapeutic effect.

## 3 Igniting immunogenic cell death in prostate cancer cells with peptides

Promoting tumor cell apoptosis is a common anti-cancer strategy. Lycosin-I, a common helical antibacterial peptide isolated from tarantula venom, features a hydrophilic α-helical structure (Tan et al., 2016). By inactivating the STAT3 pathway, high concentrations of lycosin-I induce apoptosis in PCa cells (Shen et al., 2018). This is significant because STAT3 has been shown to be overactivated and overexpressed in PCa. The STAT3 signaling pathway is closely associated with cell proliferation, differentiation, and apoptosis, and its dysregulation can lead to abnormal cell proliferation and malignant transformation (Pencik et al., 2023). Traditionally, it was believed that apoptotic cells, when rapidly phagocytosed, underwent a silent death that did not elicit an immune response. This modality of cell death was widely regarded as immunologically silent or even tolerogenic (Krysko and Vandenabeele, 2010). Therefore, considering that PCa is often classified as a “cold” tumor with a suppressed immune microenvironment, merely promoting apoptosis in PCa cells may not yield satisfactory clinical outcomes.

In recent years, a novel concept of ICD has emerged, highlighting the crucial role of the immune system in cancer therapy efficacy (Li et al., 2022). The immunogenic characteristics of ICD are primarily mediated by damage-associated molecular patterns (DAMPs), which are molecules secreted, released, or exposed on the surface of dying, stressed, or injured cells (Krysko et al., 2012). Here is an introduction to peptide-induced ICD for enhancing immunotherapy in PCa.

### 3.1 Peptide-induced ferroptosis in prostate cancer cells

The peptide TEP-FFG-CRApY leverages ferroptosis to elicit an immunogenic response (Wang et al., 2024). Ferroptosis, an iron-dependent, non-classical programmed cell death pathway, is triggered by excessive lipid peroxidation in cellular membranes when the balance between peroxidation and its repair is disrupted (Dixon and Olzmann, 2024). This peptide comprises three functional domains: a photosensitizer (TEP) for reactive oxygen species (ROS) generation, a self-assembly motif (FFG), and a GPX4-targeting sequence (CRApY). Upon internalization into tumor cells, TEP-FFG-CRApY transforms from nanoparticles to nanofibers in response to alkaline phosphatase (ALP) activity, facilitating lysosomal membrane permeabilization (LMP). This not only releases endogenous iron ions but also targets and degrades GPX4, a critical regulator of ferroptosis, under light exposure. The released iron ions further amplify ferroptosis via the Fenton reaction, generating more ROS. The immunogenic ferroptosis induced by TEP-FFG-CRApY enhances tumor immunogenicity by promoting dendritic cells (DCs) maturation and increasing T-cell infiltration into tumors (Wang et al., 2024). Notably, activated T cells secrete IFN-γ, which further downregulates the expression of SLC7A11 and SLC3A2, fostering a self-amplifying loop of ferroptosis (Wang et al., 2024). Thus, TEP-FFG-CRApY represents a novel approach to trigger immunogenic ferroptosis in “cold” tumors, such as PCa, potentiating immunotherapy by stimulating a robust anti-tumor immune response and long-term immune memory.

### 3.2 Peptide-triggered pyroptosis in prostate cancer cells

Pyroptosis stands out as another remarkable form of ICD, adept at potently activating the antitumor immune response (Loveless et al., 2021). A recent study presents a novel nano-photosensitizer, named YBS-BMS NPs-RKC, which innovatively combines pH-responsive induction of immunogenic pyroptosis with immune checkpoint blockade (ICB). This nano-photosensitizer employs a pH-responsive polymer equipped with the cell membrane-anchoring peptide RKC as its delivery vehicle. It is further encapsulated with YBS, a near-infrared-activated semiconductor polymer photosensitizer, and BMS-202, a small molecule inhibitor targeting the PD-1/PD-L1 complex. YBS-BMS NPs-RKC effectively induces immunogenic pyroptosis in PCa cells (Wang et al., 2023). During this process, the release of DAMPs and cytokines prompts the maturation of dendritic cells and the activation of tumor-specific T cells, thereby facilitating the infiltration of cytotoxic T lymphocytes (CTLs) into the TME (Wang et al., 2023).

### 3.3 Peptide modulation of autophagy in prostate cancer cells

Autophagy constitutes a highly conserved mechanism in evolution that employs lysosomes to degrade cytoplasmic components. Maintaining an invariable, foundational level of autophagy is pivotal for preserving cellular homeostasis (Mizushima and Komatsu, 2011). Nevertheless, autophagy’s involvement in cancer is notably complex; it acts as a tumor suppressor in the initial stages but facilitates tumor progression in later stages (Miller and Thorburn, 2021). Additionally, autophagy’s capacity to degrade ferritin, contingent on the expression of NCOA4 (nuclear receptor coactivator 4), has the potential to induce ferroptosis (Hou et al., 2016).

Autophagy is primarily governed by LC3 proteins, specifically microtubule-associated protein 1 light chain 3 (MAP1LC3), commonly known as LC3. These proteins are indispensable for autophagosome formation and the sequestration of cargo into these vesicles (Jacquet et al., 2021). When autophagy initiates, LC3-I undergoes a critical modification involving conjugation to phosphatidylethanolamine (PE), converting it to LC3-II, which then localizes to the lipid membrane of nascent autophagosomes (Amaravadi et al., 2011). LC3 proteins feature a unique amino acid sequence known as the LC3 interacting region (LIR), facilitating interactions with other proteins and integral participation in the autophagy machinery (Lazova et al., 2012). The LIR domain presents an attractive target for designing ligands that interact with the LC3 subfamily through a structure-based drug design strategy. Researchers have adopted a computational approach that has proven effective in identifying biologically active peptides and developing a novel peptide (pep6). By using the structure of the bacterial protein RavZ as a scaffold, they engineered pep6 to bind LC3B, resulting in the inhibition of autophagy in PCa cells (Albani et al., 2024).

Specifically, mitophagy, a well-recognized type of autophagy tailored for specific cargo, plays a pivotal role in maintaining mitochondrial quality by eliminating defective mitochondria (Meng et al., 2022). The peptide Herdegradin, which downregulates EGFR, stimulates mitophagy through the mTORC2/Akt pathway, effectively inhibiting the growth of EGFR-positive cancers, such as PCa, ultimately inducing the death of PCa cells (Katreddy et al., 2018). Recently, the tyrosine kinase activity of EGFR has become a prime therapeutic target in PCa treatment. Yet, the clinical success of EGFR tyrosine kinase inhibitors in PCa has been hampered (Xiong et al., 2020). Hence, enhancing mitophagy through diverse mechanisms offers promising new strategies to overcome drug resistance in PCa (Katreddy et al., 2018).

## 4 Revisiting immunotherapy for prostate cancer

### 4.1 Advancing immune checkpoint blockade therapy with peptide-based innovations

Immunotherapy for solid tumors primarily leverages inhibitors targeting programmed cell death-1 (PD-1)/programmed cell death ligand-1 (PD-L1) and cytotoxic T lymphocyte antigen-4 (CTLA-4), collectively known as ICB (Sun et al., 2023a).

However, a clinical trial utilizing nivolumab, a PD-1 inhibitor, in patients with metastatic castration-resistant prostate cancer (mCRPC) failed to demonstrate notable objective response rates (Topalian et al., 2012). Subsequently, clinical studies with pembrolizumab, another anti-PD-1 monoclonal antibody, in mCRPC patients also proved ineffective, indicating that monotherapy with PD-1 inhibitors in this patient population did not yield significant objective responses (Scheid et al., 2016).

The phosphorylation-mimetic peptide S249/T252, derived from the retinoblastoma protein (RB), represents a novel advancement designed to emulate the phosphorylated state of RB at specific residues, S249 and T252, which are key targets of CDK4/6 kinases (Jin et al., 2019). This peptide’s development is rooted in the groundbreaking discovery that hyperphosphorylated RB functions as a potent suppressor of NF-κB activity and PD-L1 transcription, unveiling a previously unknown tumor suppressor role of RB (Burkhart and Sage, 2008). Specifically, phosphorylated RB interacts with the NF-κB protein p65, effectively inhibiting the transcriptional activity of NF-κB (Narasimha et al., 2014). By mimicking this phosphorylated state, the S249/T252 peptide aims to disrupt the RB-NF-κB interaction and inhibit NF-κB-mediated PD-L1 transcription, thereby strengthening cancer immunity and overcoming immune evasion mechanisms triggered by both conventional and targeted therapies (Jin et al., 2019). Additionally, this peptide has demonstrated its capacity to suppress radiotherapy-induced upregulation of PD-L1 and enhance the therapeutic efficacy of radiation *in vivo*, establishing it as a promising therapeutic agent in cancer treatment (Jin et al., 2019).

Another study aimed to downregulate the expression of immune checkpoint molecules on T cells using the SIINFEKL peptide vaccine (Jeon et al., 2024). Prior research has indicated that T cells activated in the presence of toll-like receptor (TLR) stimulation, particularly TLR3 and TLR9, undergo alterations in the expression of several immune checkpoint molecules, resulting in a decrease in PD-1 expression (Hao et al., 2023). Consequently, this study employed a strategy combining TLR agonists with αCTLA-4 or αLAG-3 antibodies to bolster the efficacy of a cancer vaccine. It is noteworthy that the combination of αPD-1 with TLR3 and TLR9 agonists failed to achieve this effect, owing to the activation of Treg cells (Jeon et al., 2024). These findings hint that the optimal pairing of TLR agonists with ICB may enhance the potency of human anticancer vaccines.

Moreover, the use of ICB alone offers limited therapeutic benefits. An alternative approach to improving therapeutic outcomes is to combine ICB with other treatments (Rotte, 2019). The previously mentioned nano-photosensitizer, known as YBS-BMS NPs-RKC, is ingeniously designed to elicit pH-responsive immunogenic pyroptosis and ICB. This is achieved by integrating the PD-1/PD-L1 complex small molecule inhibitor BMS-202 with a cell membrane-anchoring peptide RKC and a near-infrared-activated semiconductor polymer photosensitizer YBS (Wang et al., 2023). This study introduces a potent, self-synergistic platform for the immunotherapy of PCa.

### 4.2 Enhancing CAR T-Cell safety with peptide-based approach

A recent Phase 1 first-in-human trial exploring PSCA-CAR T cell therapy has shown promising bioactivity and initial signs of clinical success. However, the intended dose escalation of CAR T cells was hindered by on-target toxicity, which cause cystitis. Notably, five out of 14 patients treated experienced cytokine release syndrome of Grade 1 or 2 (Dorff et al., 2024). One of the primary obstacles in CAR T cell therapy is the scarcity of a tumor-specific antigen uniquely expressed in cancer cells. Consequently, CAR T cells are frequently directed towards tumor-associated antigens (TAAs) that, despite being overexpressed in tumors, may also be found in healthy organs and tissues, posing the risk of severe or even fatal adverse effects (Albert et al., 2017). Moreover, managing CAR T cells after infusion is particularly challenging due to their unpredictable nature. They proliferate rapidly upon encountering targets and release cytokines, but there is limited understanding of how they will react individually. The absence of a safety mechanism or “switch” further increases the risk of severe or fatal side effects, such as tumor lysis syndrome and cytokine release syndrome (Jureczek et al., 2019). Therefore, there is a pressing need to improve the persistence, specificity, and safety of CAR T cell therapy to overcome these challenges and enhance its therapeutic potential.

A study has focused on pinpointing tumor-specific antigens to bolster CAR T-cell therapy. In particular, for neuroendocrine PCa (NEPC), it uncovered potential antigens by leveraging the Isoform peptides derived from RNA splicing through the Immunotherapy target Screening (IRIS) platform (Pan et al., 2023). This platform utilizes extensive tumor and normal transcriptome datasets, paired with sophisticated analytical tools, to detect tumor antigens arising from alternative splicing (AS), a mechanism where various mRNA isoforms are produced from a single gene (Baralle and Giudice, 2017). By applying the IRIS platform to NEPC RNA-seq data, numerous potential TCR targets were forecasted, with 48 epitopes being prioritized through stringent selection criteria. These epitopes demonstrated NEPC-specific expression akin to neoantigens and were encoded by microexons, presenting promising new avenues for immunotherapeutic interventions in NEPC (Pan et al., 2023).

Researchers have recently unveiled the advanced, controllable modular universal CAR T cells (UniCAR) platform technology, tailored to elevate the performance and safety profile of CAR T-cell therapy. Instead of targeting TAAs, UniCARs are engineered to recognize the human peptide sequence E5B9, also known as the UniCAR epitope, which is a fragment of the human nuclear autoantigen La/SS-B and absent in living cells (Jureczek et al., 2019). The UniCAR system is comprised of target modules (TMs) and T cells armed with UniCAR receptors (Meyer et al., 2021). These bispecific TMs possess the capability to bridge the gap between tumor cell surfaces and UniCAR-equipped T cells. Crucially, UniCAR T cells remain inactive until they encounter a corresponding TM, thereby enabling finely tuned control over their activation status (Peschke et al., 2023).

### 4.3 The potential of peptide-based redevelopment of dendritic cell vaccines

After considerable endeavors, sipuleucel-T stands alone as the immunotherapeutic agent that has yielded a significant survival benefit in a randomized Phase 3 clinical trial (Kantoff et al., 2010). Nevertheless, its withdrawal from use in Europe is a notable point. The complexity of its administration, its high cost, and supply constraints due to limited manufacturing capabilities have all contributed to its prescription challenges (Jarosławski and Toumi, 2015).

Peptides can enhance the antigenicity of DC vaccines. Researchers have crafted an innovative DC vaccine designed to bolster antigenicity by harnessing the potent synergy of MAGE-A2, a cancer/testis antigen notably upregulated in PCa, and long peptides (LPs) serving as the antigen source (Bakhshi et al., 2023). Utilizing LPs, in particular, offers several advantages compared to traditional short peptides. Notably, LPs can elicit robust responses from both CD4+ and CD8+ T cells, ensuring a more comprehensive and enduring antigen presentation. Moreover, LPs encompass a wider range of HLA types and harbor multiple epitopes, potentially sparking more vigorous and sustained antitumor activity (Corradin et al., 2010). In this study, DCs were loaded with MAGE-A2 LPs, and their capacity to stimulate T cell proliferation and induce cytotoxic T cells (CTLs) that produce IFN-γ and efficiently eliminate PCa cell lines (PC3 and LNCaP) was assessed. The findings underscore the promise of this DC vaccine as a cornerstone for advancing and enhancing PCa immunotherapy (Bakhshi et al., 2023).

Additionally, researchers have ventured into developing a Tn-MUC1 glycopeptide-DC vaccine (Scheid et al., 2016). MUC1, a glycoprotein naturally occurring on the surface of ductal epithelial cells, undergoes significant alterations during malignant transformation, including loss of polarization and overexpression in a hypoglycosylated form (Nath and Mukherjee, 2014). This altered state features truncated carbohydrates known as T or Tn tumor antigens, among which Tn-MUC1, carrying Tn carbohydrates, emerges as a promising candidate for immunotherapy. The enhanced antigenicity of glycosylated Tn-MUC1 renders it an attractive target for immune-based therapies (Scheid et al., 2016).

## 5 Peptide-based immunotherapy for prostate cancer at the development stage

### 5.1 Peptide-integrated PROTAC strategy

PROTAC (Proteolysis-Targeting Chimera) drug design constitutes an innovative approach to the development of cancer therapies, leveraging the induction of protein degradation (Paiva and Crews, 2019). It features a heterodifunctional molecule integrating two crucial ligands: one targeting and binding to the protein of interest, and another engaging with an E3 ligase, a pivotal element within the ubiquitin-proteasome system tasked with marking proteins for degradation (Zeng et al., 2021). PROTAC drugs exhibit notable advantages in drug design, enhancing specificity and efficacy while mitigating toxicity. These benefits arise from their capacity to interact over larger surfaces, thereby addressing the limitations posed by the shallow binding pockets typical of small molecule drugs (Zhang et al., 2024). Moreover, peptide-based PROTAC drugs offer even greater advantages. Thanks to their expanded interface with the target protein, peptide PROTACs demonstrate superior potency, higher selectivity, and reduced toxicity compared to low molecular weight compounds, especially when targeting proteins lacking well-defined binding pockets (Kargbo, 2019).

A recent study introduces a peptide PROTAC specifically designed to target and degrade the cysteine-histidine-rich 1 (CH1) domain of p300, a vital co-activator involved in oncogenic signaling pathways, particularly in PCa (Zhang et al., 2024). p300 holds a central position in tumor biology, influencing cancer cell survival, proliferation, metastasis, immune evasion, and drug resistance. Notably, its overexpression and hyperactivation are key drivers in the progression of CRPC (Xu et al., 2020). By employing PROTAC technology to degrade p300, the oncogenic effects are disrupted. RNA-Seq analysis of PCa cells treated with this p300-targeting peptide PROTAC revealed substantial induction of apoptosis and modulation of immune-related pathways (Zhang et al., 2024). These findings indicate that the PROTAC not only impacts cancer cell viability but also alters the immune microenvironment, presenting a promising therapeutic approach.

### 5.2 Peptide-involved epigenomic therapy

The peptide ST101 functions as a potent inhibitor of C/EBPβ (CCAAT/enhancer binding protein β), a basic leucine zipper (bZIP) transcription factor frequently upregulated or overactivated in numerous cancers (Darvishi et al., 2022). C/EBPβ plays crucial roles in regulating genes implicated in organ development, immune and inflammatory responses, such as IL-6 expression, and macrophage differentiation (Portale et al., 2024). Historically, transcription factors like C/EBPβ have posed therapeutic challenges due to their absence of enzymatic activity and well-defined small-molecule binding sites, rendering them difficult to target directly with traditional drugs (Hattori et al., 2003). However, research has demonstrated that deleting the C/EBPβ leucine zipper domain not only halts dimerization and DNA binding but also augments ubiquitination and proteasomal degradation of C/EBPβ (Bushweller, 2019). Given these insights and C/EBPβ’s emerging role as a key driver in diverse cancers, scientists aimed to develop a novel anticancer agent in the form of a C/EBPβ antagonist peptide. The resulting peptide, ST101, is tailored to target the leucine zipper domain of C/EBPβ, binding with nanomolar affinity to disrupt dimerization and DNA binding at consensus sites. Exposure to ST101 enhances ubiquitination and proteasome-dependent degradation of C/EBPβ, reducing its protein levels (Darvishi et al., 2022). *In vitro* studies have shown that ST101 promotes G0–G1 phase arrest and dose-dependent cell death in various tumor cell lines, including PCa cells, without affecting normal human immune cells (Darvishi et al., 2022).

## 6 Conclusion

PCa remains a significant global health concern, with ADT and immunotherapy facing limitations in effectively treating the disease, especially in its advanced stages. Peptide-based immunotherapy has emerged as a promising approach, offering multiple strategies to enhance the treatment of PCa. Peptides can reverse the immunosuppressive state of PCa by modulating the TME, inhibiting angiogenesis, and modulating the EMT. They also repolarize macrophages, activate NK cells, and regulate cytokines. Peptides induce ICD through ferroptosis, pyroptosis, and autophagy modulation, releasing DAMPs to activate the immune response, enhancing immunogenicity, and making cancer cells more susceptible to the immune system. They improve immunotherapeutic strategies by enhancing cancer immunity in checkpoint blockade, identifying specific tumor antigens, improving safety in CAR T-cell therapy, and boosting antigenicity in DC vaccines. Emerging strategies, such as peptide-based PROTACs and epigenomic therapy, offer new treatment options for advanced or resistant cases. However, despite the significant progress made in peptide-based immunotherapy for PCa, there are still challenges to overcome. The translation of pre-clinical findings to clinical applications requires further investigation, and issues such as peptide delivery, stability, and potential side effects need to be addressed. Additionally, more research is needed to optimize the combination of peptide-based therapies with other existing treatment modalities to achieve the best possible outcomes for patients.

In conclusion, peptide-based immunotherapy represents a highly innovative and promising area of research in PCa treatment. With continued research and development, it has the potential to revolutionize the way PCa is managed, offering new hope for patients worldwide.

## References

[B1] Abbas A. B. Lin B. Liu C. Morshed A. Hu J. Xu H. (2019). Design and Synthesis of A PD-1 binding peptide and evaluation of its anti-tumor activity. Int. J. Mol. Sci. 20 (3), 572. 10.3390/ijms20030572 30699956 PMC6386944

[B2] Achilefu S. Jimenez H. N. Dorshow R. B. Bugaj J. E. Webb E. G. Wilhelm R. R. (2002). Synthesis, *in vitro* receptor binding, and *in vivo* evaluation of fluorescein and carbocyanine peptide-based optical contrast agents. J. Med. Chem. 45 (10), 2003–2015. 10.1021/jm010519l 11985468

[B3] Albani M. Fassi E. M. A. Moretti R. M. Garofalo M. Montagnani Marelli M. Roda G. (2024). Computational design of novel cyclic peptides endowed with autophagy-inhibiting activity on cancer cell lines. Int. J. Mol. Sci. 25 (9), 4622. 10.3390/ijms25094622 38731842 PMC11083565

[B4] Albert S. Arndt C. Feldmann A. Bergmann R. Bachmann D. Koristka S. (2017). A novel nanobody-based target module for retargeting of T lymphocytes to EGFR-expressing cancer cells via the modular UniCAR platform. Oncoimmunology 6 (4), e1287246. 10.1080/2162402x.2017.1287246 28507794 PMC5414885

[B5] Amaravadi R. K. Lippincott-Schwartz J. Yin X. M. Weiss W. A. Takebe N. Timmer W. (2011). Principles and current strategies for targeting autophagy for cancer treatment. Clin. Cancer Res. 17 (4), 654–666. 10.1158/1078-0432.Ccr-10-2634 21325294 PMC3075808

[B6] Arab I. Park J. Shin J. J. Shin H. S. Suk K. Lee W. H. (2023). Macrophage lncRNAs in cancer development: long-awaited therapeutic targets. Biochem. Pharmacol. 218, 115890. 10.1016/j.bcp.2023.115890 37884197

[B7] Bakhshi P. Nourizadeh M. Sharifi L. Farajollahi M. M. Mohsenzadegan M. (2023). Development of dendritic cell loaded MAGE-A2 long peptide; a potential target for tumor-specific T cell-mediated prostate cancer immunotherapy. Cancer Cell Int. 23 (1), 270. 10.1186/s12935-023-03108-0 37951911 PMC10638778

[B8] Baralle F. E. Giudice J. (2017). Alternative splicing as a regulator of development and tissue identity. Nat. Rev. Mol. Cell Biol. 18 (7), 437–451. 10.1038/nrm.2017.27 28488700 PMC6839889

[B9] Berraondo P. Etxeberria I. Ponz-Sarvise M. Melero I. (2018). Revisiting interleukin-12 as a cancer immunotherapy agent. Clin. Cancer Res. 24 (12), 2716–2718. 10.1158/1078-0432.Ccr-18-0381 29549160

[B10] Bhatia V. Kamat N. V. Pariva T. E. Wu L. T. Tsao A. Sasaki K. (2023). Targeting advanced prostate cancer with STEAP1 chimeric antigen receptor T cell and tumor-localized IL-12 immunotherapy. Nat. Commun. 14 (1), 2041. 10.1038/s41467-023-37874-2 37041154 PMC10090190

[B11] Burkhart D. L. Sage J. (2008). Cellular mechanisms of tumour suppression by the retinoblastoma gene. Nat. Rev. Cancer 8 (9), 671–682. 10.1038/nrc2399 18650841 PMC6996492

[B12] Bushweller J. H. (2019). Targeting transcription factors in cancer - from undruggable to reality. Nat. Rev. Cancer 19 (11), 611–624. 10.1038/s41568-019-0196-7 31511663 PMC8820243

[B13] Cao Z. Kyprianou N. (2015). Mechanisms navigating the TGF-β pathway in prostate cancer. Asian J. Urol. 2 (1), 11–18. 10.1016/j.ajur.2015.04.011 29051866 PMC5645057

[B14] Cha H. R. Lee J. H. Hensel J. A. Sawant A. B. Davis B. H. Lee C. M. (2016). Prostate cancer-derived cathelicidin-related antimicrobial peptide facilitates macrophage differentiation and polarization of immature myeloid progenitors to protumorigenic macrophages. Prostate 76 (7), 624–636. 10.1002/pros.23155 26856684 PMC5551898

[B15] Chen D. Zhang X. Li Z. Zhu B. (2021). Metabolic regulatory crosstalk between tumor microenvironment and tumor-associated macrophages. Theranostics 11 (3), 1016–1030. 10.7150/thno.51777 33391518 PMC7738889

[B16] Chen S. Lu K. Hou Y. You Z. Shu C. Wei X. (2023). YY1 complex in M2 macrophage promotes prostate cancer progression by upregulating IL-6. J. Immunother. Cancer 11 (4), e006020. 10.1136/jitc-2022-006020 37094986 PMC10152059

[B17] Cheng B. Li L. Luo T. Wang Q. Luo Y. Bai S. (2024). Single-cell deconvolution algorithms analysis unveils autocrine IL11-mediated resistance to docetaxel in prostate cancer via activation of the JAK1/STAT4 pathway. J. Exp. Clin. Cancer Res. 43 (1), 67. 10.1186/s13046-024-02962-8 38429845 PMC10905933

[B18] Cho W. K. Spille J. H. Hecht M. Lee C. Li C. Grube V. (2018). Mediator and RNA polymerase II clusters associate in transcription-dependent condensates. Science 361 (6400), 412–415. 10.1126/science.aar4199 29930094 PMC6543815

[B19] Chow A. Perica K. Klebanoff C. A. Wolchok J. D. (2022). Clinical implications of T cell exhaustion for cancer immunotherapy. Nat. Rev. Clin. Oncol. 19 (12), 775–790. 10.1038/s41571-022-00689-z 36216928 PMC10984554

[B20] Collazo J. Zhu B. Larkin S. Martin S. K. Pu H. Horbinski C. (2014). Cofilin drives cell-invasive and metastatic responses to TGF-β in prostate cancer. Cancer Res. 74 (8), 2362–2373. 10.1158/0008-5472.Can-13-3058 24509905 PMC4488067

[B21] Corradin G. Kajava A. V. Verdini A. (2010). Long synthetic peptides for the production of vaccines and drugs: a technological platform coming of age. Sci. Transl. Med. 2 (50), 50rv3. 10.1126/scitranslmed.3001387 20861510

[B22] Culp M. B. Soerjomataram I. Efstathiou J. A. Bray F. Jemal A. (2020). Recent global patterns in prostate cancer incidence and mortality rates. Eur. Urol. 77 (1), 38–52. 10.1016/j.eururo.2019.08.005 31493960

[B23] Daquinag A. C. Dadbin A. Snyder B. Wang X. Sahin A. A. Ueno N. T. (2017). Non-glycanated decorin is a drug target on human adipose stromal cells. Mol. Ther. Oncolytics 6, 1–9. 10.1016/j.omto.2017.05.003 28607949 PMC5458115

[B24] Darvishi E. Ghamsari L. Leong S. F. Ramirez R. Koester M. Gallagher E. (2022). Anticancer activity of ST101, A novel antagonist of CCAAT/enhancer binding protein β. Mol. Cancer Ther. 21 (11), 1632–1644. 10.1158/1535-7163.Mct-21-0962 36121385 PMC9630826

[B25] Di Donato M. Bilancio A. D'Amato L. Claudiani P. Oliviero M. A. Barone M. V. (2015). Cross-talk between androgen receptor/filamin A and TrkA regulates neurite outgrowth in PC12 cells. Mol. Biol. Cell 26 (15), 2858–2872. 10.1091/mbc.E14-09-1352 26063730 PMC4571344

[B26] Di Donato M. Giovannelli P. Barone M. V. Auricchio F. Castoria G. Migliaccio A. (2021a). A small peptide targeting the ligand-induced androgen receptor/filamin a interaction inhibits the invasive phenotype of prostate cancer cells. Cells 11 (1), 14. 10.3390/cells11010014 35011576 PMC8750472

[B27] Di Donato M. Zamagni A. Galasso G. Di Zazzo E. Giovannelli P. Barone M. V. (2021b). The androgen receptor/filamin A complex as a target in prostate cancer microenvironment. Cell Death Dis. 12 (1), 127. 10.1038/s41419-021-03402-7 33500395 PMC7838283

[B28] Dixon S. J. Olzmann J. A. (2024). The cell biology of ferroptosis. Nat. Rev. Mol. Cell Biol. 25 (6), 424–442. 10.1038/s41580-024-00703-5 38366038 PMC12187608

[B29] Dorff T. B. Blanchard M. S. Adkins L. N. Luebbert L. Leggett N. Shishido S. N. (2024). PSCA-CAR T cell therapy in metastatic castration-resistant prostate cancer: a phase 1 trial. Nat. Med. 30 (6), 1636–1644. 10.1038/s41591-024-02979-8 38867077 PMC11186768

[B30] Figueiredo M. L. Letteri R. Chan-Seng D. Kumar S. Rivera-Cruz C. M. Emrick T. S. (2021). Reengineering tumor microenvironment with sequential interleukin delivery. Bioeng. (Basel) 8 (7), 90. 10.3390/bioengineering8070090 PMC8301035 34209203

[B31] Figueiredo Neto M. Liu S. Salameh J. W. Yokota H. Figueiredo M. L. (2020). Interleukin-27 gene delivery targeting IL-6rα-expressing cells as a stress response therapy. Int. J. Mol. Sci. 21 (3), 1108. 10.3390/ijms21031108 32046108 PMC7038084

[B32] Gascard P. Tlsty T. D. (2016). Carcinoma-associated fibroblasts: orchestrating the composition of malignancy. Genes Dev. 30 (9), 1002–1019. 10.1101/gad.279737.116 27151975 PMC4863733

[B33] Gasparri A. M. Sacchi A. Basso V. Cortesi F. Freschi M. Rrapaj E. (2019). Boosting interleukin-12 antitumor activity and synergism with immunotherapy by targeted delivery with isoDGR-tagged nanogold. Small 15 (45), e1903462. 10.1002/smll.201903462 31523920

[B34] Goel H. L. Moro L. King M. Teider N. Centrella M. McCarthy T. L. (2006). Beta1 integrins modulate cell adhesion by regulating insulin-like growth factor-II levels in the microenvironment. Cancer Res. 66 (1), 331–342. 10.1158/0008-5472.Can-05-2588 16397247

[B35] Gonzalez D. M. Medici D. (2014). Signaling mechanisms of the epithelial-mesenchymal transition. Sci. Signal 7 (344), re8. 10.1126/scisignal.2005189 25249658 PMC4372086

[B36] Gupta D. Gupta V. Nath D. Miglani C. Mandal D. Pal A. (2023). Stimuli-responsive self-assembly disassembly in peptide amphiphiles to endow block-co-fibers and tunable piezoelectric response. ACS Appl. Mater Interfaces 15 (21), 25110–25121. 10.1021/acsami.2c05469 35767722

[B37] Hanold L. E. Fulton M. D. Kennedy E. J. (2017). Targeting kinase signaling pathways with constrained peptide scaffolds. Pharmacol. Ther. 173, 159–170. 10.1016/j.pharmthera.2017.02.014 28185915 PMC5407947

[B38] Hao Y. Li H. Ge X. Liu Y. Yin J. Li X. (2023). Site-specific nanoswitch circumventing immune resistance via activating TLR and inhibiting PD-L1/PD-1 axis. J. Control Release 361, 64–76. 10.1016/j.jconrel.2023.07.048 37532143

[B39] Hattori T. Ohoka N. Inoue Y. Hayashi H. Onozaki K. (2003). C/EBP family transcription factors are degraded by the proteasome but stabilized by forming dimer. Oncogene 22 (9), 1273–1280. 10.1038/sj.onc.1206204 12618752

[B40] Heidegger I. Fotakis G. Offermann A. Goveia J. Daum S. Salcher S. (2022). Comprehensive characterization of the prostate tumor microenvironment identifies CXCR4/CXCL12 crosstalk as a novel antiangiogenic therapeutic target in prostate cancer. Mol. Cancer 21 (1), 132. 10.1186/s12943-022-01597-7 35717322 PMC9206324

[B41] Hou W. Xie Y. Song X. Sun X. Lotze M. T. Zeh H. J. 3rd (2016). Autophagy promotes ferroptosis by degradation of ferritin. Autophagy 12 (8), 1425–1428. 10.1080/15548627.2016.1187366 27245739 PMC4968231

[B42] Hynes R. O. (2009). The extracellular matrix: not just pretty fibrils. Science 326 (5957), 1216–1219. 10.1126/science.1176009 19965464 PMC3536535

[B43] Jacquet M. Guittaut M. Fraichard A. Despouy G. (2021). The functions of Atg8-family proteins in autophagy and cancer: linked or unrelated? Autophagy 17 (3), 599–611. 10.1080/15548627.2020.1749367 32255730 PMC8032235

[B44] Jarosławski S. Toumi M. (2015). Sipuleucel-T (Provenge(®))-Autopsy of an innovative paradigm change in cancer treatment: why a single-product biotech company failed to capitalize on its breakthrough invention. BioDrugs 29 (5), 301–307. 10.1007/s40259-015-0140-7 26403092

[B45] Jaynes J. M. Sable R. Ronzetti M. Bautista W. Knotts Z. Abisoye-Ogunniyan A. (2020). Mannose receptor (CD206) activation in tumor-associated macrophages enhances adaptive and innate antitumor immune responses. Sci. Transl. Med. 12 (530), eaax6337. 10.1126/scitranslmed.aax6337 32051227 PMC7832040

[B46] Jeon D. Hill E. Moseman J. E. McNeel D. G. (2024). Combining toll-like receptor agonists with immune checkpoint blockade affects antitumor vaccine efficacy. J. Immunother. Cancer 12 (5), e008799. 10.1136/jitc-2024-008799 38702146 PMC11086196

[B47] Jia H. Yang H. Xiong H. Luo K. Q. (2023). NK cell exhaustion in the tumor microenvironment. Front. Immunol. 14, 1303605. 10.3389/fimmu.2023.1303605 38022646 PMC10653587

[B48] Jin X. Ding D. Yan Y. Li H. Wang B. Ma L. (2019). Phosphorylated RB promotes cancer immunity by inhibiting NF-κB activation and PD-L1 expression. Mol. Cell 73 (1), 22–35.e6. 10.1016/j.molcel.2018.10.034 30527665 PMC8968458

[B49] Jones E. Pu H. Kyprianou N. (2009). Targeting TGF-beta in prostate cancer: therapeutic possibilities during tumor progression. Expert Opin. Ther. Targets 13 (2), 227–234. 10.1517/14728220802705696 19236240

[B50] Jureczek J. Bergmann R. Berndt N. Koristka S. Kegler A. Puentes-Cala E. (2019). An oligo-His-tag of a targeting module does not influence its biodistribution and the retargeting capabilities of UniCAR T cells. Sci. Rep. 9 (1), 10547. 10.1038/s41598-019-47044-4 31332252 PMC6646371

[B51] Kang J. La Manna F. Bonollo F. Sampson N. Alberts I. L. Mingels C. (2022). Tumor microenvironment mechanisms and bone metastatic disease progression of prostate cancer. Cancer Lett. 530, 156–169. 10.1016/j.canlet.2022.01.015 35051532

[B52] Kantoff P. W. Higano C. S. Shore N. D. Berger E. R. Small E. J. Penson D. F. (2010). Sipuleucel-T immunotherapy for castration-resistant prostate cancer. N. Engl. J. Med. 363 (5), 411–422. 10.1056/NEJMoa1001294 20818862

[B53] Kargbo R. B. (2019). Treatment of prostate cancers and kennedy's disease by PROTAC-androgen receptor degradation. ACS Med. Chem. Lett. 10 (5), 701–702. 10.1021/acsmedchemlett.9b00115 31097985 PMC6512008

[B54] Katreddy R. R. Bollu L. R. Su F. Xian N. Srivastava S. Thomas R. (2018). Targeted reduction of the EGFR protein, but not inhibition of its kinase activity, induces mitophagy and death of cancer cells through activation of mTORC2 and Akt. Oncogenesis 7 (1), 5. 10.1038/s41389-017-0021-7 29358623 PMC5833766

[B55] Kawada M. Inoue H. Masuda T. Ikeda D. (2006). Insulin-like growth factor I secreted from prostate stromal cells mediates tumor-stromal cell interactions of prostate cancer. Cancer Res. 66 (8), 4419–4425. 10.1158/0008-5472.Can-05-4239 16618768

[B56] Krysko D. V. Garg A. D. Kaczmarek A. Krysko O. Agostinis P. Vandenabeele P. (2012). Immunogenic cell death and DAMPs in cancer therapy. Nat. Rev. Cancer 12 (12), 860–875. 10.1038/nrc3380 23151605

[B57] Krysko D. V. Vandenabeele P. (2010). Clearance of dead cells: mechanisms, immune responses and implication in the development of diseases. Apoptosis 15 (9), 995–997. 10.1007/s10495-010-0524-6 20645005

[B58] Kwon J. T. W. Bryant R. J. Parkes E. E. (2021). The tumor microenvironment and immune responses in prostate cancer patients. Endocr. Relat. Cancer 28 (8), T95–t107. 10.1530/erc-21-0149 34128831 PMC8345898

[B59] Lazova R. Camp R. L. Klump V. Siddiqui S. F. Amaravadi R. K. Pawelek J. M. (2012). Punctate LC3B expression is a common feature of solid tumors and associated with proliferation, metastasis, and poor outcome. Clin. Cancer Res. 18 (2), 370–379. 10.1158/1078-0432.Ccr-11-1282 22080440 PMC4825867

[B60] Li C. Xu X. Wei S. Jiang P. Xue L. Wang J. (2021). Tumor-associated macrophages: potential therapeutic strategies and future prospects in cancer. J. Immunother. Cancer 9 (1), e001341. 10.1136/jitc-2020-001341 33504575 PMC8728363

[B61] Li J. Ruffell B. (2022). Cytokines drive prostate cancer lineage plasticity. Immunity 55 (10), 1761–1763. 10.1016/j.immuni.2022.09.011 36223725

[B62] Li M. Li M. Yang Y. Liu Y. Xie H. Yu Q. (2020). Remodeling tumor immune microenvironment via targeted blockade of PI3K-γ and CSF-1/CSF-1R pathways in tumor associated macrophages for pancreatic cancer therapy. J. Control Release 321, 23–35. 10.1016/j.jconrel.2020.02.011 32035193

[B63] Li Z. Lai X. Fu S. Ren L. Cai H. Zhang H. (2022). Immunogenic cell death activates the tumor immune microenvironment to boost the immunotherapy efficiency. Adv. Sci. (Weinh) 9 (22), e2201734. 10.1002/advs.202201734 35652198 PMC9353475

[B64] Liu H. Zhao Z. Zhang L. Li Y. Jain A. Barve A. (2019). Discovery of low-molecular weight anti-PD-L1 peptides for cancer immunotherapy. J. Immunother. Cancer 7 (1), 270. 10.1186/s40425-019-0705-y 31640814 PMC6805442

[B65] Loveless R. Bloomquist R. Teng Y. (2021). Pyroptosis at the forefront of anticancer immunity. J. Exp. Clin. Cancer Res. 40 (1), 264. 10.1186/s13046-021-02065-8 34429144 PMC8383365

[B66] Mao C. Ding Y. Xu N. (2021). A double-edged sword role of cytokines in prostate cancer immunotherapy. Front. Oncol. 11, 688489. 10.3389/fonc.2021.688489 34868907 PMC8635015

[B67] Mehta K. Patel K. Parikh R. A. (2017). Immunotherapy in genitourinary malignancies. J. Hematol. Oncol. 10 (1), 95. 10.1186/s13045-017-0457-4 28434403 PMC5402074

[B68] Meng Y. Qiu L. Zeng X. Hu X. Zhang Y. Wan X. (2022). Targeting CRL4 suppresses chemoresistant ovarian cancer growth by inducing mitophagy. Signal Transduct. Target Ther. 7 (1), 388. 10.1038/s41392-022-01253-y 36481655 PMC9731993

[B116] Meyer J. E. Loff S. Dietrich J. Spehr J. Jurado Jiménez G. von Bonin M. (2021). Evaluation of switch-mediated costimulation in trans on universal CAR-T cells (UniCAR) targeting CD123-positive AML. Oncoimmunology 10 (1), 1945804. 10.1080/2162402x.2021.1945804 34290907 PMC8274446

[B69] Miller D. R. Thorburn A. (2021). Autophagy and organelle homeostasis in cancer. Dev. Cell 56 (7), 906–918. 10.1016/j.devcel.2021.02.010 33689692 PMC8026727

[B70] Mizushima N. Komatsu M. (2011). Autophagy: renovation of cells and tissues. Cell 147 (4), 728–741. 10.1016/j.cell.2011.10.026 22078875

[B71] Monieri M. Rainone P. Sacchi A. Gori A. Gasparri A. M. Coliva A. (2023). A stapled chromogranin A-derived peptide homes in on tumors that express αvβ6 or αvβ8 integrins. Int. J. Biol. Sci. 19 (1), 156–166. 10.7150/ijbs.76148 36594095 PMC9760430

[B72] Muttenthaler M. King G. F. Adams D. J. Alewood P. F. (2021). Trends in peptide drug discovery. Nat. Rev. Drug Discov. 20 (4), 309–325. 10.1038/s41573-020-00135-8 33536635

[B73] Narasimha A. M. Kaulich M. Shapiro G. S. Choi Y. J. Sicinski P. Dowdy S. F. (2014). Cyclin D activates the Rb tumor suppressor by mono-phosphorylation. Elife 3, e02872. 10.7554/eLife.02872 24876129 PMC4076869

[B74] Nardelli F. Ghitti M. Quilici G. Gori A. Luo Q. Berardi A. (2019). A stapled chromogranin A-derived peptide is a potent dual ligand for integrins αvβ6 and αvβ8. Chem. Commun. (Camb) 55 (98), 14777–14780. 10.1039/c9cc08518a 31755501

[B75] Nath S. Mukherjee P. (2014). MUC1: a multifaceted oncoprotein with a key role in cancer progression. Trends Mol. Med. 20 (6), 332–342. 10.1016/j.molmed.2014.02.007 24667139 PMC5500204

[B76] Nguyen P. L. Alibhai S. M. Basaria S. D'Amico A. V. Kantoff P. W. Keating N. L. (2015). Adverse effects of androgen deprivation therapy and strategies to mitigate them. Eur. Urol. 67 (5), 825–836. 10.1016/j.eururo.2014.07.010 25097095

[B77] Ochiya T. Takenaga K. Asagiri M. Nakano K. Satoh H. Watanabe T. (2015). Efficient inhibition of tumor angiogenesis and growth by a synthetic peptide blocking S100A4-methionine aminopeptidase 2 interaction. Mol. Ther. Methods Clin. Dev. 2, 15008. 10.1038/mtm.2015.8 26029719 PMC4445002

[B78] Ohishi T. Abe H. Sakashita C. Saqib U. Baig M. S. Ohba S. I. (2020). Inhibition of mitochondria ATP synthase suppresses prostate cancer growth through reduced insulin-like growth factor-1 secretion by prostate stromal cells. Int. J. Cancer 146 (12), 3474–3484. 10.1002/ijc.32959 32144767

[B79] Özdemir B. C. Pentcheva-Hoang T. Carstens J. L. Zheng X. Wu C. C. Simpson T. R. (2014). Depletion of carcinoma-associated fibroblasts and fibrosis induces immunosuppression and accelerates pancreas cancer with reduced survival. Cancer Cell 25 (6), 719–734. 10.1016/j.ccr.2014.04.005 24856586 PMC4180632

[B80] Paiva S. L. Crews C. M. (2019). Targeted protein degradation: elements of PROTAC design. Curr. Opin. Chem. Biol. 50, 111–119. 10.1016/j.cbpa.2019.02.022 31004963 PMC6930012

[B81] Pan Y. Phillips J. W. Zhang B. D. Noguchi M. Kutschera E. McLaughlin J. (2023). IRIS: discovery of cancer immunotherapy targets arising from pre-mRNA alternative splicing. Proc. Natl. Acad. Sci. U. S. A. 120 (21), e2221116120. 10.1073/pnas.2221116120 37192158 PMC10214192

[B82] Pencik J. Philippe C. Schlederer M. Atas E. Pecoraro M. Grund-Gröschke S. (2023). STAT3/LKB1 controls metastatic prostate cancer by regulating mTORC1/CREB pathway. Mol. Cancer 22 (1), 133. 10.1186/s12943-023-01825-8 37573301 PMC10422794

[B83] Peschke J. C. Bergmann R. Mehnert M. Gonzalez Soto K. E. Loureiro L. R. Mitwasi N. (2023). FLT3-directed UniCAR T-cell therapy of acute myeloid leukaemia. Br. J. Haematol. 202 (6), 1137–1150. 10.1111/bjh.18971 37460273

[B84] Portale F. Carriero R. Iovino M. Kunderfranco P. Pandini M. Marelli G. (2024). C/EBPβ-dependent autophagy inhibition hinders NK cell function in cancer. Nat. Commun. 15 (1), 10343. 10.1038/s41467-024-54355-2 39609420 PMC11604937

[B85] Rotte A. (2019). Combination of CTLA-4 and PD-1 blockers for treatment of cancer. J. Exp. Clin. Cancer Res. 38 (1), 255. 10.1186/s13046-019-1259-z 31196207 PMC6567914

[B86] Ryan C. J. Haqq C. M. Simko J. Nonaka D. F. Chan J. M. Weinberg V. (2007). Expression of insulin-like growth factor-1 receptor in local and metastatic prostate cancer. Urol. Oncol. 25 (2), 134–140. 10.1016/j.urolonc.2006.07.019 17349528

[B87] Saha A. Kolonin M. G. DiGiovanni J. (2023). Obesity and prostate cancer - microenvironmental roles of adipose tissue. Nat. Rev. Urol. 20 (10), 579–596. 10.1038/s41585-023-00764-9 37198266

[B88] Scheid E. Major P. Bergeron A. Finn O. J. Salter R. D. Eady R. (2016). Tn-MUC1 DC vaccination of rhesus macaques and a phase I/II trial in patients with nonmetastatic castrate-resistant prostate cancer. Cancer Immunol. Res. 4 (10), 881–892. 10.1158/2326-6066.Cir-15-0189 27604597 PMC5331878

[B89] Shen H. Xie Y. Ye S. He K. Yi L. Cui R. (2018). Spider peptide toxin lycosin-I induces apoptosis and inhibits migration of prostate cancer cells. Exp. Biol. Med. (Maywood) 243 (8), 725–735. 10.1177/1535370218772802 29763387 PMC6378508

[B90] Sioud M. Westby P. Olsen J. K. Mobergslien A. (2015). Generation of new peptide-Fc fusion proteins that mediate antibody-dependent cellular cytotoxicity against different types of cancer cells. Mol. Ther. Methods Clin. Dev. 2, 15043. 10.1038/mtm.2015.43 26605373 PMC4632835

[B91] Song H. Yang P. Huang P. Zhang C. Kong D. Wang W. (2019). Injectable polypeptide hydrogel-based co-delivery of vaccine and immune checkpoint inhibitors improves tumor immunotherapy. Theranostics 9 (8), 2299–2314. 10.7150/thno.30577 31149045 PMC6531311

[B92] Su F. Ahn S. Saha A. DiGiovanni J. Kolonin M. G. (2019). Adipose stromal cell targeting suppresses prostate cancer epithelial-mesenchymal transition and chemoresistance. Oncogene 38 (11), 1979–1988. 10.1038/s41388-018-0558-8 30361686 PMC6417957

[B93] Subudhi S. K. Vence L. Zhao H. Blando J. Yadav S. S. Xiong Q. (2020). Neoantigen responses, immune correlates, and favorable outcomes after ipilimumab treatment of patients with prostate cancer. Sci. Transl. Med. 12 (537), eaaz3577. 10.1126/scitranslmed.aaz3577 32238575

[B94] Sun H. Dong Y. Feijen J. Zhong Z. (2018). Peptide-decorated polymeric nanomedicines for precision cancer therapy. J. Control Release 290, 11–27. 10.1016/j.jconrel.2018.09.029 30290243

[B95] Sun L. Kienzler J. C. Reynoso J. G. Lee A. Shiuan E. Li S. (2023a). Immune checkpoint blockade induces distinct alterations in the microenvironments of primary and metastatic brain tumors. J. Clin. Invest 133 (17), e169314. 10.1172/jci169314 37655659 PMC10471177

[B96] Sun Y. Cronin M. F. Mendonça M. C. P. Guo J. O'Driscoll C. M. (2023b). M2pep-Modified cyclodextrin-siRNA nanoparticles modulate the immunosuppressive tumor microenvironment for prostate cancer therapy. Mol. Pharm. 20 (11), 5921–5936. 10.1021/acs.molpharmaceut.3c00769 37874541 PMC10630955

[B97] Takeda A. Hamano S. Yamanaka A. Hanada T. Ishibashi T. Mak T. W. (2003). Cutting edge: role of IL-27/WSX-1 signaling for induction of T-bet through activation of STAT1 during initial Th1 commitment. J. Immunol. 170 (10), 4886–4890. 10.4049/jimmunol.170.10.4886 12734330

[B98] Takenaga K. Ochiya T. Endo H. (2021). Inhibition of the invasion and metastasis of mammary carcinoma cells by NBD peptide targeting S100A4 via the suppression of the Sp1/MMP-14 axis. Int. J. Oncol. 58 (3), 397–408. 10.3892/ijo.2021.5173 33650647 PMC7864152

[B99] Tan H. Luo W. Wei L. Chen B. Li W. Xiao L. (2016). Quantifying the distribution of the stoichiometric composition of anticancer peptide lycosin-I on the lipid membrane with single molecule spectroscopy. J. Phys. Chem. B 120 (12), 3081–3088. 10.1021/acs.jpcb.5b12618 26937786

[B100] Topalian S. L. Hodi F. S. Brahmer J. R. Gettinger S. N. Smith D. C. McDermott D. F. (2012). Safety, activity, and immune correlates of anti-PD-1 antibody in cancer. N. Engl. J. Med. 366 (26), 2443–2454. 10.1056/NEJMoa1200690 22658127 PMC3544539

[B101] Topalian S. L. Hodi F. S. Brahmer J. R. Gettinger S. N. Smith D. C. McDermott D. F. (2019). Five-year survival and correlates among patients with advanced melanoma, renal cell carcinoma, or non-small cell lung cancer treated with nivolumab. JAMA Oncol. 5 (10), 1411–1420. 10.1001/jamaoncol.2019.2187 31343665 PMC6659167

[B102] Tsang B. Pritišanac I. Scherer S. W. Moses A. M. Forman-Kay J. D. (2020). Phase separation as a missing mechanism for interpretation of disease mutations. Cell 183 (7), 1742–1756. 10.1016/j.cell.2020.11.050 33357399

[B103] Tse B. W. Russell P. J. Lochner M. Förster I. Power C. A. (2011). IL-18 inhibits growth of murine orthotopic prostate carcinomas via both adaptive and innate immune mechanisms. PLoS One 6 (9), e24241. 10.1371/journal.pone.0024241 21935389 PMC3174151

[B104] Van Aarsen L. A. Leone D. R. Ho S. Dolinski B. M. McCoon P. E. LePage D. J. (2008). Antibody-mediated blockade of integrin alpha v beta 6 inhibits tumor progression *in vivo* by a transforming growth factor-beta-regulated mechanism. Cancer Res. 68 (2), 561–570. 10.1158/0008-5472.Can-07-2307 18199553

[B105] Wala J. Nguyen P. Pomerantz M. (2023). Early treatment intensification in metastatic hormone-sensitive prostate cancer. J. Clin. Oncol. 41 (20), 3584–3590. 10.1200/jco.23.00723 37267579 PMC10325768

[B106] Wang H. He Z. Gao Y. Feng D. Wei X. Huang Y. (2023). Dual-pronged attack: pH-driven membrane-anchored NIR dual-type nano-photosensitizer excites immunogenic pyroptosis and sequester immune checkpoint for enhanced prostate cancer photo-immunotherapy. Adv. Sci. (Weinh) 10 (28), e2302422. 10.1002/advs.202302422 37544896 PMC10558672

[B107] Wang H. Jiao D. Feng D. Liu Q. Huang Y. Hou J. (2024). Transformable supramolecular self-assembled peptides for cascade self-enhanced ferroptosis primed cancer immunotherapy. Adv. Mater 36 (21), e2311733. 10.1002/adma.202311733 38339920

[B108] Winkler J. Abisoye-Ogunniyan A. Metcalf K. J. Werb Z. (2020). Concepts of extracellular matrix remodelling in tumour progression and metastasis. Nat. Commun. 11 (1), 5120. 10.1038/s41467-020-18794-x 33037194 PMC7547708

[B109] Wise G. J. Marella V. K. Talluri G. Shirazian D. (2000). Cytokine variations in patients with hormone treated prostate cancer. J. Urol. 164 (3 Pt 1), 722–725. 10.1097/00005392-200009010-00024 10953133

[B110] Xiang X. Wang J. Lu D. Xu X. (2021). Targeting tumor-associated macrophages to synergize tumor immunotherapy. Signal Transduct. Target Ther. 6 (1), 75. 10.1038/s41392-021-00484-9 33619259 PMC7900181

[B111] Xiong Y. Yuan L. Chen S. Xu H. Peng T. Ju L. (2020). WFDC2 suppresses prostate cancer metastasis by modulating EGFR signaling inactivation. Cell Death Dis. 11 (7), 537. 10.1038/s41419-020-02752-y 32678075 PMC7366654

[B112] Xu S. Fan L. Jeon H. Y. Zhang F. Cui X. Mickle M. B. (2020). p300-Mediated acetylation of histone demethylase JMJD1A prevents its degradation by ubiquitin ligase STUB1 and enhances its activity in prostate cancer. Cancer Res. 80 (15), 3074–3087. 10.1158/0008-5472.Can-20-0233 32522824 PMC7415556

[B113] Zeng S. Huang W. Zheng X. Liyan C. Zhang Z. Wang J. (2021). Proteolysis targeting chimera (PROTAC) in drug discovery paradigm: recent progress and future challenges. Eur. J. Med. Chem. 210, 112981. 10.1016/j.ejmech.2020.112981 33160761

[B114] Zhang D. Ma B. Liu D. Wu W. Zhou T. Gao Y. (2024). Discovery of a peptide proteolysis-targeting chimera (PROTAC) drug of p300 for prostate cancer therapy. EBioMedicine 105, 105212. 10.1016/j.ebiom.2024.105212 38954976 PMC11261775

[B115] Zhang Y. Zhang Z. (2020). The history and advances in cancer immunotherapy: understanding the characteristics of tumor-infiltrating immune cells and their therapeutic implications. Cell Mol. Immunol. 17 (8), 807–821. 10.1038/s41423-020-0488-6 32612154 PMC7395159

